# Personalized Approaches to Stroke: One Step Forward for Functional Recovery of Stroke Patients

**DOI:** 10.3390/jpm12050822

**Published:** 2022-05-19

**Authors:** Won Hyuk Chang

**Affiliations:** Department of Physical and Rehabilitation Medicine, Center for Prevention and Rehabilitation, Heart Vascular and Stroke Institute, Samsung Medical Center, Sungkyunkwan University School of Medicine, Seoul 06351, Korea; wh.chang@samsung.com

**Keywords:** personalized medicine, stroke, precision medicine, recovery, rehabilitation

## Abstract

Recent advances in diagnoses, management, and rehabilitation have had a significant impact to reduce mortality and functional recovery in stroke patients. In spite of these medical advances, many stroke survivors still suffer from significant disabilities. Stroke is a complex disease caused by a combination of multiple risk factors. Therefore, personalized medicine is more important than any other field to overcome the limitations of current stroke management and rehabilitation. It is necessary to apply accurate evaluation for functions and a personalized approach in consideration of various characteristics of each stroke patient to improve function. The objective of this Special Issue is to inform the recent scientific knowledge, current limitations, and challenges for an individually tailored strategy in the areas of diagnosis, treatment, and rehabilitation of stroke. A multidisciplinary approach and research will be strongly encouraged for personalized medicine in the field of stroke treatment and rehabilitation.

Stroke is a leading cause of morbidity and mortality in the world [1]. Recent advances in diagnoses, management, and rehabilitation have had a significant impact on reducing mortality and functional recovery in stroke patients [2,3]. In spite of these medical advances, many stroke survivors still suffer from significant disability [4], and disability after stroke remains a burden to patients, caregivers, and society [5]. Most of the studies on stroke treatment and rehabilitation so far have been proposed through the analysis of average stroke patients [3]. Stroke is a complex disease caused by a combination of multiple risk factors [6]. Therefore, personalized medicine is more important than any other field in order to overcome the limitations of current stroke treatment. Recently, several review papers discuss the importance and necessity of a personalized approach to stroke treatment and rehabilitation [3,6,7].

It is necessary to apply accurate evaluation for functions and personalized treatment methods in consideration of the various characteristics of each stroke patient. In order to propose a new personalized treatment, the check should be performed first for the common treatment methods that have been widely used. Stretching has been most widely used in the physical management of post-stroke spasticity [8]. However, no conclusive evidence was analyzed on the effectiveness of stretching for post-stroke spasticity [9]. This meta-analysis proposed an individual stretching method based on stroke patients’ characteristics [9]. Zhang et al. [10] reported that endovascular coil embolization for aneurysmal subarachnoid hemorrhage (SAH) showed a higher risk than surgical clipping. In addition, they also reported that other independent poor prognostic factors were male sex, older age, hypertension, congestive heart failure, diabetes, and previous stroke for vasospasm and re-stroke. Through this paper [10], it would be helpful for an individual approach in selecting a treatment method for patients with aneurysmal SAH.

The understanding of the functional recovery mechanism should also be preceded by the development of novel personalized therapy in stroke patients. Kim and Kang [11] reported the changes in structural connectivity of the bilateral hemispheres according to the time following the stroke, and Lee et al. [12] reported that the conditional role of the corticocerebellar tract depends on corticospinal tract status for upper extremity recovery in stroke patients. Park et al. [13] suggested that the alterations in the spinothalamic tract and superior thalamic radiation could be the pathogenesis of central post-stroke pain. Ko et al. [14] reported that one of the pathophysiologies of post-stroke spasticity might be corticoreticular pathway injury. These insightful articles [11,12,13,14] could provide useful information about personalized rehabilitative strategies in stroke patients.

Rapid advances in robotics, virtual reality, and artificial intelligence have led to the use of new technologies in the areas of stroke treatment and rehabilitation. Major et al. [15] reported the study on the practical use of robotic systems, and Lee et al. [16] reported that a deep model in a 3D convolutional neural network using video data recorded by a smartphone could classify dependence in ambulation in stroke patients. Luque-Moreno et al. [17] suggested that the intervention with virtual reality could be a feasible treatment in stroke patients. These studies [15,16,17] are expected to be used as new measurements and advanced treatment methods in stroke patients.

New advanced treatment methods have been reported for stroke patients. However, such new treatment methods cannot be effective for all stroke patients. Therefore, it is very important to study which patients are suitable for the new treatment method. In order to apply individual treatment, research on the mechanism of action of a new treatment method is also essential. Lee et al. [18] reported the change of cortical activity by high-definition transcranial direct current stimulation (HD-tDCS) and Chang et al. [19] showed the potential mechanisms of Cerebrolysin for improving motor function in stroke patients [19]. Recent studies suggested appropriate indications for stem cell therapy [20] and transcranial direct current stimulation (tDCS) [21]. Each age and stroke duration from onset to treatment could be considered for personalized mesenchymal stem cell therapy for ischemic stroke patients [20]. Catechol-O-methyltransferase polymorphism could be used to select the proper candidate of tDCS for dysphagia in stroke patients [21]. This article is meaningful in that genetic factors might be considered in stroke treatment. These reports [18,19,20,21] might be used as references for personalized stroke therapy in the future. 

Personalized medicine is a growing field in diagnosing, managing and predicting stroke and will enable clinicians to develop individually tailored care plans for stroke patients [3,6,7,22]. All papers published in this Special Issue will be able to contribute to the advance of personalized medicine in strokes [9,10,11,12,13,14,15,16,17,18,19,20,21]. The objective of this editorial is to stimulate any research on personalized medicine for the evaluation and treatment of stroke patients.

It is expected that personalized medicine for the evaluation and treatment of stroke patients will continue to develop in the future. However, there are still many hurdles to overcome to reach personalized medicine for stroke patients. Through the papers published in this Special Issue [9,10,11,12,13,14,15,16,17,18,19,20,21], it was found that a number of factors must be considered, and a new and diverse approach was also required for personalized medicine. Therefore, a multidisciplinary approach and research are strongly encouraged for the personalized medicine of stroke treatment and rehabilitation.
